# Mutant LV^476-7^AA of A-subunit of *Enterococcus hirae* V_1_-ATPase: High affinity of A_3_B_3_ complex to DF axis and low ATPase activity

**DOI:** 10.1186/2193-1801-2-689

**Published:** 2013-12-27

**Authors:** Jahangir Alam, Ichiro Yamato, Satoshi Arai, Shinya Saijo, Kenji Mizutani, Yoshiko Ishizuka-Katsura, Noboru Ohsawa, Takaho Terada, Mikako Shirouzu, Shigeyuki Yokoyama, So Iwata, Yoshimi Kakinuma, Takeshi Murata

**Affiliations:** Department of Biological Science and Technology, Tokyo University of Science, 6-3-1 Niijuku, Katsushika-ku, Tokyo, 125-8585 Japan; Department of Genetic Engineering and Biotechnology, School of Life Sciences, Shahjalal University of Science and Technology, Sylhet, 3114 Bangladesh; Department of Chemistry, Graduate School of Science, Chiba University, 1-33 Yayoi-choInage, Chiba, 263-8522 Japan; RIKEN SPring-8 Center, 1-1-1 Kouto, Sayo, Hyogo, 679-5148 Japan; Structural Biology Research Center, Photon Factory, Institute of Materials Structure Science, High Energy Accelerator Research Organization (KEK), Tsukuba, Ibaraki, 305-0801 Japan; RIKEN Systems and Structural Biology Center, 1-7-22 Suehiro-cho, Tsurumi, Yokohama, 230-0045 Japan; Division of Structural and Synthetic Biology, RIKEN Center for Life Science Technologies, 1-7-22 Suehiro-cho, Tsurumi, Yokohama, 230-0045 Japan; RIKEN Structural Biology Laboratory, 1-7-22 Suehiro-cho, Tsurumi, Yokohama, 230-0045 Japan; Department of Cell Biology, Faculty of Medicine, Kyoto University, Yoshidakonoe-cho, Sakyo-ku, Kyoto, 606-8501 Japan; Laboratory of Molecular Physiology and Genetics, Faculty of Agriculture, Ehime University, 3-5-7 Tarumi, Matsuyama, Ehime, 790-8566 Japan; JST, PRESTO, 1-33 Yayoi-cho, Inage, Chiba, 263-8522 Japan

**Keywords:** Site-directed mutation, Reconstitution, Catalytic domain, ATPase assay, Surface plasmon resonance, *Enterococcus hirae*

## Abstract

Vacuolar ATPase (V-ATPase) of *Enterococcus hirae* is composed of a soluble functional domain V_1_ (A_3_B_3_DF) and an integral membrane domain V_o_ (ac), where V_1_ and V_o_ domains are connected by a central stalk, composed of D-, F-, and d-subunits; and two peripheral stalks (E- and G-subunits). We identified 120 interacting residues of A_3_B_3_ heterohexamer with D-subunit in DF heterodimer in the crystal structures of A_3_B_3_ and A_3_B_3_DF. In our previous study, we reported 10 mutants of *E. hirae* V_1_-ATPase, which showed lower binding affinities of DF with A_3_B_3_ complex leading to higher initial specific ATPase activities compared to the wild-type. In this study, we identified a mutation of A-subunit (LV^476-7^AA) at its C-terminal domain resulting in the A_3_B_3_ complex with higher binding affinities for wild-type or mutant DF heterodimers and lower initial ATPase activities compared to the wild-type A_3_B_3_ complex, consistent with our previous proposal of reciprocal relationship between the ATPase activity and the protein-protein binding affinity of DF axis to the A_3_B_3_ catalytic domain of *E. hirae* V-ATPase. These observations suggest that the binding of DF axis at the contact region of A_3_B_3_ rotary ring is relevant to its rotation activity.

## Introduction

Vacuolar ATPase (V-ATPase) functions as a proton pump in the acidic organelles and plasma membranes of eukaryotic cells and bacteria (Forgac 2007; Stevens & Forgac 1997). This acidic environment is essential for such processes as receptor-mediated endocytosis, intracellular targeting of lysosomal enzymes, protein processing and degradation (Forgac 2007). ATPases possess an overall similar structure composed of a catalytic portion (F_1_-, V_1_-, or A_1_-ATPase) and a membrane-embedded ion-transporting portion (F_o_-, V_o_-, or A_o_-ATPase), and have a similar reaction mechanism as rotary motors (Forgac 2007).

**Figure 1:**
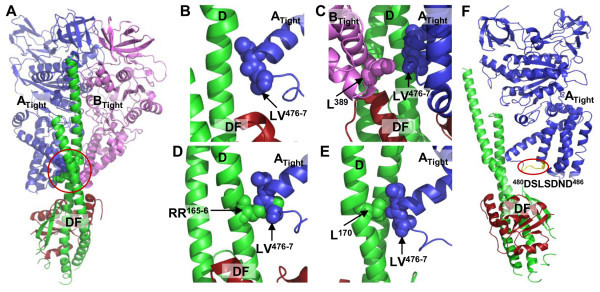
**Positions of the critical contact residues of A- and/or B-subunits with DF complex in the structure of** ***E. hirae*** **V**_**1**_ **-ATPase (Arai et al.** 2013 **; Saijo et al.** 2011 **). (A)** The side-viewed ribbon representation of the “tight” form of A- (*tv_blue*) and B-subunit (*violet*) together with DF (*tv_green* and *firebrick*, respectively) complex. Spheres (in *red circle*) indicate the selected residues (Figure 1B-E) for mutation of the corresponding subunits. **(B)** The closer view of the critical contact residue LV^476-7^ (*tv_blue spheres*) of the A-subunit with DF complex. **(C)** The closer view of the critical contact residues (LV^476-7^ (*tv_blue spheres*) of A-subunit and L^389^ (*violet spheres*) of B-subunit) of the “tight” form of A- and B-subunits together with DF complex. **(D)** The closer view of the “tight” form of A-subunit together with DF complex showing the critical contact residues; LV^476-7^ (*tv_blue spheres*) of A-subunit and RR^165-6^ (*tv_green spheres*) of D-subunit. **(E)** The closer view of the “tight” form of A-subunit together with DF complex showing the critical contact residues; LV^476-7^ (*tv_blue spheres*) of A-subunit and L^170^ (*tv_green spheres*) of D-subunit. **(F)** The “tight” form of A-subunit (*tv_blue*) together with DF (*tv_green* and *firebrick*, respectively) complex showing the ^480^DSLSDND^486^ sequence of A-subunit (*yellow* in *red circle*), probably corresponding to the DELSEED sequence of F-ATPase (Mnatsakanyan et al. 2011; Nakanishi-Matsui & Futai 2008).

V-ATPases are found in bacteria, such as *Thermus thermophilus* and *Enterococcus hirae. T. thermophilus* V-ATPase physiologically functions as an ATP synthase (Lee et al. 2010), whereas, *E. hirae* V-ATPase is not an ATP synthase and instead acts as a primary ion pump similar to eukaryotic V-ATPases, which transports Na^+^ or Li^+^ instead of H^+^ (Murata et al. 2000 Murata et al. 2005b, 2008; Furutani et al. 2011; Mizutani et al. 2011). The enzyme is composed of nine subunits having amino acid sequences that are homologous to those of the corresponding subunits of eukaryotic V-ATPases (Murata et al. 1997, 2005a; Yamamoto et al. 2008; Zhou et al. 2011). Amino acid sequences and subunit structures are more similar to eukaryotic V-ATPases than to ATP synthases of F- and V-ATPases. The V_1_ domain of V-ATPase is composed of a hexameric arrangement of alternating A- and B-subunits responsible for ATP binding and hydrolysis (Murata et al. 1999) and the V_o_ domain is composed of the 16-kDa c-subunits and an a-subunit in which rotational energy is converted to drive Na^+^ translocation (Furutani et al. 2011; Mizutani et al. 2011). The V_1_ and V_o_ domains are connected by a central stalk (composed of D-, F-, and d-subunits) and 2 peripheral stalks (composed of E- and G-subunits of V_1_) (Murata et al. 2005a; & Yamamoto et al. 2008). During ATP hydrolysis, the central axis (the DFd complex) attached on the membrane c-ring rotates inside the hexagonally arranged A_3_B_3_ complex, which causes ion pumping at the interface between the c-ring and a-subunit (Murata et al. 2008). Single molecular studies of *E. hirae* V_1_-ATPase showed 120° steps of rotation without any substeps driven by ATP hydrolysis, as commonly seen with F_1_-ATPase (Minagawa et al. 2013). Previously, we reported the reconstitution and purification of A_3_B_3_ and A_3_B_3_DF of *E. hirae* V-ATPase (Arai et al. 2009) and solved the crystal structures of DF, A_3_B_3_, and A_3_B_3_DF (Arai et al. 2013; Saijo et al. 2011). Crystal structures of these complexes suggest the existence of 120 polar and nonpolar (van der Waals) interactions between the A_3_B_3_ and DF complexes and ATP hydrolysis seems to be stimulated by the approach of a conserved arginine residue (Arai et al. 2013). Recently, we reported the mutational effects of 10 interacting residues at the conserved C-terminal domain (near the ^480^DSLSDND^486^ sequence of A-subunit (Figure 1F), probably corresponding to the DELSEED sequence of F-ATPase (Mnatsakanyan et al. 2011; Nakanishi-Matsui & Futai 2008)) of A- and B-subunits with the residues of D-subunit, showing higher initial ATPase activities and lower binding affinities compared to the wild-type (Alam et al. 2013).

In this study, we constructed another mutant (LV^476-7^AA) (Figure 1B) neighboring to the ^480^DSLSDND^486^ sequence (Figure 1F) of A-subunit. We reconstituted the V_1_ domains containing different mutational combinations including wild-type and previous mutations (Alam et al. 2013) (Figure 1C-E) and measured the initial ATPase activities and binding affinities of those V_1_-ATPases that showed higher binding affinities and lower initial ATPase activities than that of the wild-type.

## Results

### Reconstitution and purification of A(LV^476-7^AA)_3_B_3_ and A(LV^476-7^AA)_3_B(L^389^A)_3_ heterohexamers

**Figure 2:**
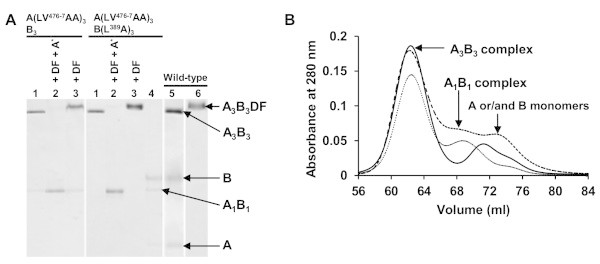
**Basic native-PAGE pattern and gel-filtration profiles for the reconstitution and purification of A**_**3**_ **B**_**3**_ **complexes from A- and B-monomers. (A)** Basic native-PAGE pattern stained with CBB R-250. *Lane 1*, gel-filtration purified mutant A_3_B_3_ heterohexamers reconstituted with 200 μM AMP-PNP; *lane 2*, reconstituted mutant A_3_B_3_ heterohexamers with DF heterodimer in the presence of 2 mM AMP-PNP; *lane 3*, reconstituted mutant A_3_B_3_ heterohexamers with DF heterodimer without nucleotides; *lane 4*, purified A(LV^476-7^AA)_3_B(L^389^A)_3_ heterohexamer after storage at 4 ºC for 20 days; *lane 5*, wild-type A_3_B_3_ with A and B monomers; and *lane 6*, wild-type A_3_B_3_DF. One μg of protein was loaded in each lane. A* indicates addition of AMP-PNP. **(B)** Gel-filtration profiles for the purification of mutant A_3_B_3_ heterohexamers reconstituted from A and B monomers. *Dotted line,* A(LV^476-7^AA)_3_B_3_; *dashed line,* A(LV^476-7^AA)_3_B(L^389^A)_3_; and *solid line,* wild-type A_3_B_3_. Gel-filtration was performed as described in “Materials and methods”. Mixture of total 6.1 mg (mixing ratio of A- and B-subunits were A:B = 65:52 (1:1 molar ratio)) samples in buffer A were loaded in Superose 6 pg gel-filtration column (500 × 16 mm ID) (GE Healthcare) and eluted with the same buffer. Purified A_3_B_3_ complex by gel-filtration was examined on basic native-PAGE as lane 1 (as shown in Figure 2A).

From the crystal structures of A_3_B_3_ and A_3_B_3_DF (Arai et al. 2013), we identified LV^476-7^ of A-subunit and L^389^ of B-subunit located closely with the interacting D-subunit in the “tight” form (A_CR_-B_CR_ pair) of V_1_-ATPase (Figure 1C). So, in this study we used the previously constructed L^389^A mutant of B-subunit (Alam et al. 2013) to reconstitute A(LV^476-7^AA)_3_B(L^389^A)_3_ heterohexamer. A(LV^476-7^AA) monomer showed a very low efficiency of complex formation with either wild-type B or B(L^389^A) monomer in the presence of 2 mM ATP, distinct from wild-type (Arai et al. 2009), but we found that A(LV^476-7^AA) showed efficient reconstitution of A(LV^476-7^AA)_3_B_3_ and A(LV^476-7^AA)_3_B(L^389^A)_3_ heterohexamers in the presence of 200 μM AMP-PNP (analogue of ATP) instead of 2 mM ATP (Figure 2A, *lane 1*). Both mutant heterohexamers were purified by gel-filtration chromatography (Figure 2A-B). These complexes seemed stable in the absence of nucleotides, although A(LV^476-7^AA)_3_B_3_ and A(LV^476-7^AA)_3_B(L^389^A)_3_ heterohexamers showed lower stability than the wild-type A_3_B_3_; in native PAGE, a band at position of A(LV^476-7^AA)_1_B_1_ or A(LV^476-7^AA)_1_B(L^389^A)_1_ complex was observed after a few days storage at -80˚C and A(LV^476-7^AA)_3_B(L^389^A)_3_ heterohexamer dissociated into monomers after a few weeks storage at 4°C (Figure 2A, *lane 4* (after 2–3 weeks stored at 4°C)).

### Reconstitution of A_3_B_3_DF (V_1_ domain) complex

A(LV^476-7^AA)_3_B_3_ and A(LV^476-7^AA)_3_B(L^389^A)_3_ heterohexamers formed V_1_ domains (A(LV^476-7^AA)_3_B_3_DF and A(LV^476-7^AA)_3_B(L^389^A)_3_DF complex, respectively) of V-ATPase with DF heterodimer (Figure 2A). When A(LV^476-7^AA)_3_B_3_ and A(LV^476-7^AA)_3_B(L^389^A)_3_ heterohexamers were incubated with DF heterodimer in the absence of nucleotides, both heterohexamers formed catalytic domains, A(LV^476-7^AA)_3_B_3_DF and A(LV^476-7^AA)_3_B(L^389^A)_3_DF, respectively (Figure 2A), showing the similar extent of reconstitution of V_1_ domains as the wild-type. The crystal structures of A_3_B_3_ and A_3_B_3_DF (Arai et al. 2013) suggested that LV^476-7^ of A-subunit closely reside to the RR^165-6^ and L^170^ of D-subunit when V_1_ is in its “tight” form (Figure 1D-E). So, we reconstituted two additional mutant V_1_ domains; A(LV^476-7^AA)_3_B_3_D(RR^165-6^AA)F and A(LV^476-7^AA)_3_B_3_D(L^170^N)F, showing the similar reconstitution efficiencies in native-PAGE like as the wild-type (data not shown), which indicates similar structural integrity of the purified mutant V_1_-ATPases.

### Biochemical properties of the reconstituted mutant catalytic domains

**Figure 3:**
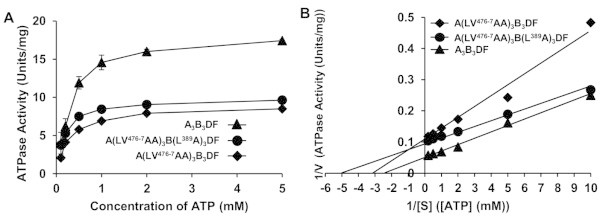
**ATPase activities of mutant A**_**3**_ **B**_**3**_ **DF (V**_**1**_ **) complexes (containing mutation in A- and/or B-subunits) of** ***E. hirae*** **V-ATPase at various ATP concentrations.** ATPase assay was started by the addition of 4 μg proteins. Experimental details were described in “Materials and methods”. **(A)** ATPase activities depending on the various concentrations of ATP were shown. **(B)** Lineweaver-Burk plots of the ATPase activities for the calculation of *K*_m_ and *V*_max_. *Filled diamonds*, A(LV^476-7^AA)_3_B_3_DF; *filled circles*, A(LV^476-7^AA)_3_B(L^389^A)_3_DF; and *filled triangles*, wild-type A_3_B_3_DF.

Initial specific activities of the reconstituted A(LV^476-7^AA)_3_B_3_DF and A(LV^476-7^AA)_3_B(L^389^A)_3_DF complexes (7.9 units/mg and 9.1 units/mg, respectively, Figure 3A, Table 1) were about half of the wild-type (16.0 units/mg). The *K*_m_ values for ATP of A(LV^476-7^AA)_3_B_3_DF and A(LV^476-7^AA)_3_B(L^389^A)_3_DF complexes were not so much different (0.45 mM and 0.27 mM, respectively) from that of wild-type (*K*_m_ = 0.4 mM) (Figure 3B) and reconstituted A(LV^476-7^AA)_3_B_3_D(RR^165-6^AA)F and A(LV^476-7^AA)_3_B_3_D(L^170^N)F complexes showed almost similar initial specific activities (15.5 units/mg and 13.0 units/mg, respectively, Table 1) like as the wild-type(16.0 units/mg).

**Table 1:** **Summary of ATPase activities of V**_**1**_ **complexes containing mutant A**_**3**_ **B**_**3**_ **heterohexamers and wild-type/mutant DF heterodimers and the binding affinities of those mutants measured by SPR assay**

Protein	Initial specific activity (units/mg)^*^	***K***_D_(nM) (using mutant A_3_B_3_as ligand and mutant/wild-type DF as analyte)
**A(LV** ^**476-7**^ **AA)**_**3**_ **B**_**3**_ **DF**	7.9 ± 0.3	1.1 ± 0.2
**A(LV** ^**476-7**^ **AA)**_**3**_ **B(L** ^**389**^ **A)**_**3**_ **DF**	9.1 ± 0.2	1.2 ± 0.1
**A(LV** ^**476-7**^ **AA)**_**3**_ **B**_**3**_ **D(RR** ^**165-6**^ **AA)F**	15.5 ± 1.4	50.9 ± 8.4
**A(LV** ^**476-7**^ **AA)**_**3**_ **B**_**3**_ **D(L** ^**170**^ **N)F**	13.0 ± 0.3	1.4 ± 0.3
**Wild-type A**_**3**_ **B**_**3**_ **DF**	16.0 ± 0.2	1.6 ± 0.1

ATPase activities of the reconstituted mutant A_3_B_3_DF’s were measured using ATP regenerating system (Alam et al. 2013; & Murata et al. 2001). ATPase assay was started by the addition of 4 μg proteins. For SPR assays, different concentrations of analyte wild-type/mutant DF heterodimer were injected onto the sensor chip Ni-NTA surface having immobilized mutant A_3_B_3_ heterohexamers. Reconstituted mutant A_3_B_3_ heterohexamers and wild-type/mutant DF heterodimer were diluted in running buffer (20 mM MES-Tris, pH 6.5; 150 mM NaCl; 50 μM EDTA-Na; 0.005% polyoxyethylene (20) sorbitol monolaurate). Experimental details were described in “Materials and methods”.

^*^“Initial specific activity” was calculated by measuring the specific activity during the first minute of the assay (starting from the 16^th^ second) after adding proteins.

A(LV^476-7^AA)_3_B_3_ heterohexamer showed higher binding affinity (dissociation constant, *K*_D_ 1.1 nM, Table 1) to wild-type DF heterodimer than the wild-type A_3_B_3_ complex (*K*_D_ 1.6 nM, Table 1) and other mutational combinations of A_3_B_3_ and DF complexes showed binding affinities between these ranges (Table 1). These findings (Table 1) indicate that lower ATPase activity (probably the rotation speed) is due to the tight binding of the DF axis to the rotary ring A_3_B_3_. There was one exception to this rule; the mutant A(LV^476-7^AA)_3_B_3_ heterohexamer showed very low binding affinity for D(RR^165-6^AA)F (Table 1) with similar initial specific activity like as the wild-type.

## Discussion

In this study, we selected the amino acid residues for mutation at the contact regions of C-terminal domain of the A-subunit (Figure 1B-E) (Arai et al. 2013). During ATP hydrolysis by V_1_-ATPase, D-subunit rotates inside the hexagonally arranged A_3_B_3_ complex and comes in contact to the residues of A- and/or B-subunits, which probably correspond to the conserved DELSEED-loop of the β-subunit of F-ATPase (Mnatsakanyan et al. 2011; Nakanishi-Matsui & Futai 2008). From structure and sequence analysis of *E. hirae* V-ATPase, we considered the residues ^480^DSLSDND^486^ of A-subunit (Figure 1F) is the corresponding loop of DELSEED of F-ATPase. We substituted the amino acids leucine and valine with alanine because of its stable helix forming tendency (Rohl et al. 1999). We demonstrated that purified mutant monomers- A(LV^476-7^AA) and B(L^389^A) were capable to form heterohexamers, A(LV^476-7^AA)_3_B_3_ and A(LV^476-7^AA)_3_B(L^389^A)_3_, like as the wild-type (Figure 2A) (Arai et al. 2009). They formed catalytic domains (V_1_-ATPases) with wild-type/mutant DF heterodimers as the similar extent of the wild-type. These mutant V_1_-ATPases were functionally active and showed different initial specific activities depending on the nature of the amino acid substituted. Hydrophilic/polar arginine (Arakawa et al. 2007) or strong hydrophobic/non-polar amino acids like as valine or leucine may form stronger interaction with other polar or non-polar amino acids, respectively, in proteins. From the crystal structures of *E. hirae* V_1_-ATPase (Arai et al. 2013; Saijo et al. 2011), we found that C-terminal residues of A-subunit, LV^476-7^ (of A_CR_-B_CR_ pair in “tight” form) are in close contact with R^165^, L^170^ (Figure 1D-E) and some other residues of D-subunit. Also, a residue at the corresponding region of B-subunit-L^389^ (of A_CR_-B_CR_ pair) is closely located near LV^476-7^ residues of A-subunit (Figure 1C). We expected that these closely residing amino acids should have strong interactions with each other which should be influential to the rotation activity. So, we selected those neighboring residues of ^480^DSLSDND^486^ sequence of A-subunit (Figure 1F) and changed all these arginine, leucine, and valine to relatively low hydrophobic and helix forming alanine. We assumed that by substitution with alanine, the binding affinities should decrease leading to higher ATPase activities. But unexpectedly all the mutant A_3_B_3_DFs containing A(LV^476-7^AA) mutation showed similar to lower initial specific activities with higher binding affinities than those of the wild-type (Figure 3A, Table 1). From this observation, we speculated that the substitution of two larger amino acids (leucine and valine) by smaller amino acid (alanine) may have resulted in a slight conformational difference of A-subunit which might be suitable for closer contact with D-subunit. Substitution effects of all the DELSEED loop residues with alanine have been already reported to resulting in the similar unidirectional rotation with kinetic parameters comparable to those of the wild-type F_1_ (Tanigawara et al. 2012). Substitution of each residue and all five acidic residues in the DELSEED sequence with alanine resulted in the similar torque as the wild-type (Hara et al. 2000). Moreover, recently Usukura et al. (Usukura et al. 2012) reported that deletion of one or two turns in the α-helix at the DELSEED region in the C-terminal domain of catalytic β subunit at the rotor/central stalk contact region of *Bacillus* PS3 F_1_-ATPase reduced the torque as well as ATPase activity to about half of the wild-type. Their result indicated that the mutants with the shortened loop can synthesize ATP and produce normal torque (Usukura et al. 2012) and ATPase activity. It would be interesting if we obtain 3D structures of these mutant A_3_B_3_DF or α_3_β_3_γ to see the structural difference from wild-type and to estimate the interaction strength with DF or γ subunit. Considering their report (Usukura et al. 2012), we have tried to obtain several deletion mutants of A-subunit at the contact site with D-subunit, but they could not be purified, probably due to their instability. When we replaced leucine (L) to asparagine (N) (in case of D mutant, D(L^170^N)), we found almost similar ATPase activity as the wild-type and small difference of binding affinity correlating with initial specific activities (Table 1).

In our previous study, we observed relationship of higher ATPase activity with lower binding affinity of D(RR^165-6^ AA) mutant with wild-type A_3_B_3_ complex (Alam et al. 2013). When this mutant formed V_1_ complex with A(LV^476-7^AA)_3_B_3_, it showed very low binding affinity with high ATPase activity (compared to A(LV^476-7^AA)_3_B_3_DF, Table 1), not higher than the wild-type A_3_B_3_DF; this low ATPase activity may be because of the dissociation of some DF heterodimer during ATPase assay owing to the low affinity, consistent with the expectation that RR^165-6^ of D-subunit is closely located to LV^476-7^ of A-subunit in “tight” form (A_CR_-B_CR_ pair) of our crystal structures (Figure 1D) (Arai et al. 2013; Saijo et al. 2011). V_1_-ATPase combined with A(LV^476-7^AA) and D(L^170^N)F mutants gave compensating effect showing nearly similar specific activities and binding affinities to those of each single mutant, suggesting the substantial interaction between A(LV^476-7^) and D(L^170^) (Table 1), consistent with our crystal structures (Figure 1E) (Arai et al. 2013; Saijo et al. 2011).

## Materials and methods

### Expression and purification of wild-type/mutant A- and B-subunits, and DF subcomplex

Synthesized DNA fragments corresponding to the *A* and *B* genes with optimal codon usage for an *Escherichia coli* expression system were cloned into the plasmid vector pET23d (Arai et al. 2009). Mutant A-subunit was constructed using the wild-type *A* gene in the plasmid as the template for PCR-generated mutation. Wild-type/mutant A- and B-subunits were independently expressed in *E. coli* BL21 (DE3) in modified-Davis Mingioli-Casamino Acid (m-DM-CA) medium (Mogi & Anraku, 1984) at 30°C as described in a previous report (Arai et al. 2009). Proteins were purified essentially according to the reported method (Arai et al. 2009; Alam et al. 2013) using Ni-Sepharose 6 fast flow (GE Healthcare) and gel-filtration chromatography (Superose 6 pg column (500 × 16 mm ID) (GE Healthcare)). Purified proteins were analyzed by sodium dodecyl sulfate-polyacrylamide gel electrophoresis (SDS-PAGE) and subsequently stained with CBB R-250. Purified proteins were concentrated by ultrafiltration using Amicon Ultra-4 30 K filters (Millipore Corporation, USA) and stored at -80°C until use. From 1 liter culture, the amount of purified A(LV^476-7^AA) proteins obtained was 20 mg. We also tried to obtain a deletion mutant ΔRLV^475-7^ of A-subunit at the C-terminal domain and also a double mutant VL^388-9^AA of B-subunit locating at the conserved region of D-subunit, but VL^388-9^AA could not be expressed and ΔRLV^475-7^ was not purified due to its instability.

To synthesize the wild-type/mutant DF complex, an *E. coli* cell-free protein expression system was used, as described elsewhere (Kigawa et al. 2004), by using plasmids coding genes for D- and F-subunits. The expressed protein was purified as previously described (Yamamoto et al. 2008). Mutagenesis of D-subunit was performed using the QuikChange site-directed mutagenesis kit (Agilent Technologies) as described (Arai et al. 2013).

### Reconstitution of mutant catalytic domains (A_3_B_3_DF/V_1_) from reconstituted wild-type/mutant A_3_B_3_ heterohexamers and synthesized DF heterodimers

The A_3_B_3_ complex was reconstituted from the purified A- and B-subunits with slight modification of previous method (Arai et al. 2009; Alam et al. 2013) using 200 μM AMP-PNP instead of 2 mM ATP. Briefly, the purified A- and B-subunits (3.4 and 2.7 mg of A- and B-subunits, respectively, at a 1:1 molar ratio) were mixed and the volume was adjusted to 4 mL with buffer A (20 mM MES-Tris, pH 6.5; 50 mM KCl; 10% glycerol; 5 mM MgSO_4_; 0.1 mM DTT). The protein mixture was then incubated on ice for 1 h in the presence of 200 μM AMP-PNP and afterward concentrated to 100 μL by ultrafiltration using Amicon Ultra-4 30 K filters (Millipore Corporation, USA). Then, 4 mL of buffer A with AMP-PNP was added to dilute the protein solution, and the solution was concentrated again to 100 μL. This dilution/concentration process was repeated thrice without adding AMP-PNP. The A_3_B_3_ heterohexamer was finally purified using a Superose 6 pg column (500 × 16 mm ID) (GE Healthcare). Complex formation was confirmed by using basic native-PAGE as previously described (Alam et al. 2013). For the reconstitution of wild-type and mutant catalytic V_1_ domain (A_3_B_3_DF), purified wild-type/mutant A_3_B_3_ heterohexamers and synthesized DF heterodimer were incubated on ice for 1 hour mixing at a 1:5 molar ratio (Arai et al. 2009; Alam et al. 2013) and the formation of the complexes were checked by using basic native-PAGE (Arai et al. 2009; Alam et al. 2013).

### ATPase assay of the reconstituted mutant A_3_B_3_DF complexes

Initial ATPase activities of the reconstituted A_3_B_3_DF were measured by ATP regenerating system (Alam et al. 2013; Murata et al. 2001). The assay mixture contained various concentrations of ATP, 2.5 mM phosphoenolpyruvate, 50 μg/mL pyruvate kinase, 50 μg/mL lactate dehydrogenase, and 0.2 mM β-NADH (dipotassium salt) in 1 mL of assay buffer (25 mM MES-Tris (pH 6.5), 4 mM MgSO_4_, 10% glycerol). The reaction was initiated by adding 4 μg proteins. The rate of ATP hydrolysis was monitored at 25°C in terms of the rate of oxidation of NADH, as determined by the decrease in absorbance at 340 nm. Specific activities were calculated as units/mg proteins, with 1 unit of ATPase activity being defined as hydrolysis of 1 μmol ATP/min. Initial ATPase activity was calculated by measuring the specific activity during the first minute (starting from the 16^th^ second) after adding the proteins. The measurement was repeated three times and averaged and the standard deviation was calculated. *K*_m_ and *V*_max_ were calculated by fitting the averaged values as straight lines in Lineweaver-Burk plots.

### Measurement of real-time binding affinity using surface plasmon resonance (SPR)

The binding affinity of DF complex to the reconstituted A_3_B_3_ complex was measured by SPR analysis on a Biacore T100 instrument (GE Healthcare Bio-sciences, AB, Sweden) as described previously (Alam et al. 2013; Arai et al. 2013; Saijo et al. 2011). The Biacore Ni-NTA sensor chip (GE Healthcare Bio-sciences) was activated with 0.5 μM NiCl_2_ as described by the manufacturer. The reconstituted A_3_B_3_ complex was immobilized at a concentration of 35 μg/mL in running buffer (20 mM MES-Tris, pH 6.5; 150 mM NaCl; 50 μM EDTA-Na; 0.005% polyoxyethylene (20) sorbitol monolaurate), passing through the Biacore flow cell at a rate of 10 μL/min. A flow cell containing no protein served as a negative control. Different concentrations of DF complex were prepared as analyte in the running buffer. The obtained sensorgrams were evaluated using Biacore T100 evaluation software. The equilibrium constant for dissociation, *K*_D_, were obtained using the Langmuir binding model (1:1 binding model).

### Chemicals/reagents and other experimental protocols

Protein concentration was determined by DC Protein Assay Kit (Bio-Rad Laboratories) using bovine serum albumin as the standard. To check the purified proteins, SDS-PAGE was performed according to Laemmli (Laemmli 1970), and stained with Coomassie brilliant blue (CBB) R-250. Restriction enzymes were purchased from Nippon Gene Japan, New England BioLabs Japan, and Wako Pure Chem. Indust., Ltd. All other chemicals were of analytical grade and purchased from Sigma-Aldrich Japan KK or Wako Pure Chem. Indust., Ltd.

## Abbreviations

CBB: Coomassie brilliant blue
DTT: Dithiothreitol
EDTA: Ethylenediamine-N,N,N’,N’-tetraacetic acid
IPTG: Isopropyl thio-β-galactoside
KD: Dissociation constant
MES: 2-(N-morpholino) ethanesulphonic acid
m-DM-CA: Modified-Davis Mingioli-Casamino acid
SPR: Surface plasmon resonance
TEV: Tobacco etch virus
V-ATPase: Vacuolar ATPase.
